# Supplementary material for: Association between oral microbiome and seven types of cancers in East Asian population: a two-sample Mendelian randomization analysis
Source: Front Mol Biosci. 2023 Nov 21;10:1327893. doi: 10.3389/fmolb.2023.1327893 (PMC10702768; doi:10.3389/fmolb.2023.1327893)
Supplement: Supplementary file 1 [file DataSheet1.ZIP › Supplementary Materials/MR plots for tongue/tongue═╝/Ovarian cancer/pheno.1308_to_ovarian cancer_forest.pdf]

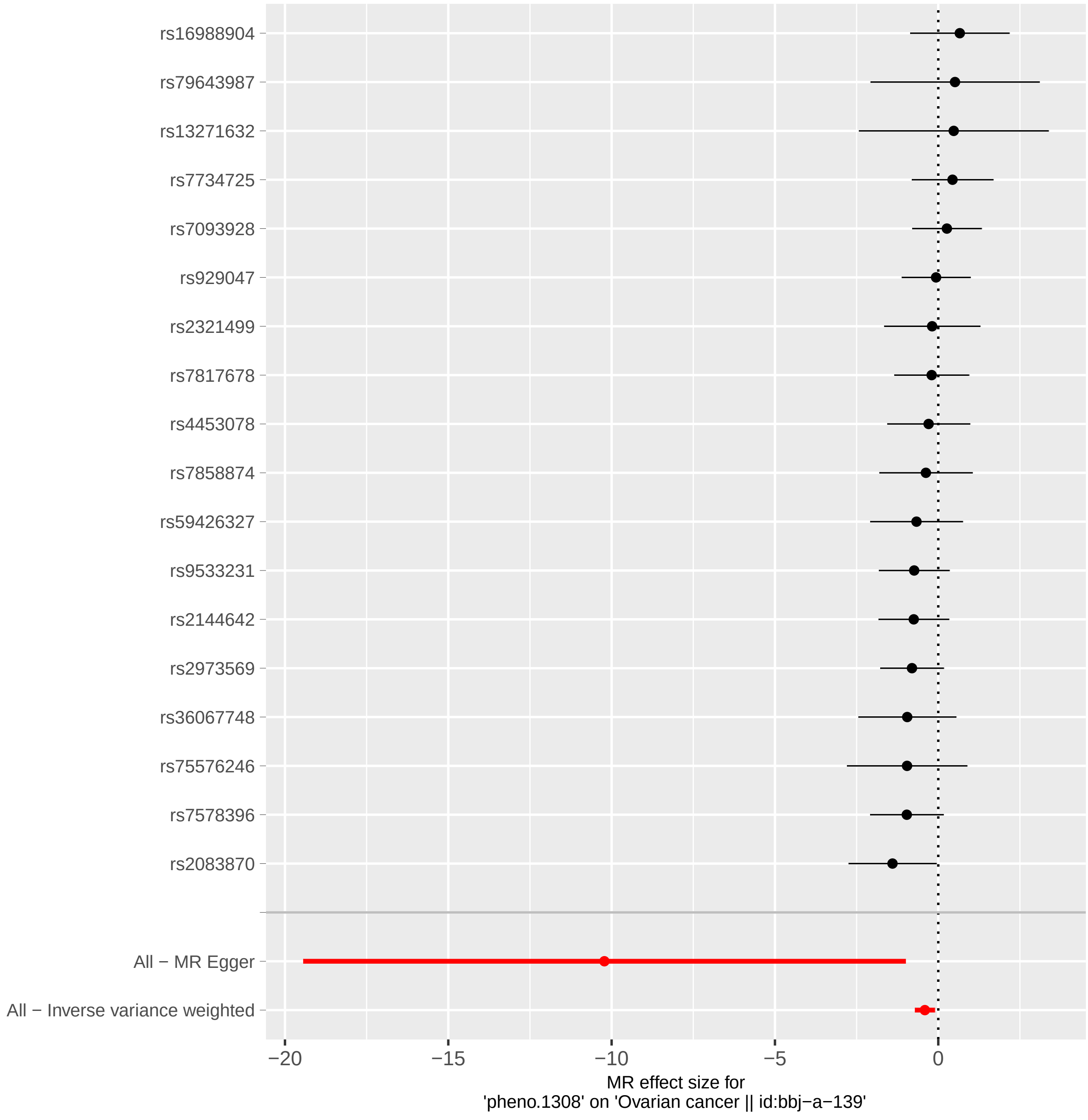

rs16988904
rs79643987
rs13271632
rs7734725
rs7093928
rs929047
rs2321499
rs7817678
rs4453078
rs7858874
rs59426327
rs9533231
rs2144642
rs2973569
rs36067748
rs75576246
rs7578396
rs2083870
All − MR Egger
All − Inverse variance weighted
−20
−15
−10
−5
0
MR effect size for
'pheno.1308' on 'Ovarian cancer || id:bbj−a−139'
